# Copper Nanoparticles Enable Multi-Compartment Cuproenzyme Metalation in Cellular Models of Copper Deficiency

**DOI:** 10.1021/acschembio.6c00564

**Published:** 2026-08-13

**Authors:** Harman Kaur, John Soukar, Nikita Gudekar, Vinit C. Shanbhag, Michael J. Petris, Akhilesh K. Gaharwar, Vishal M. Gohil

**Affiliations:** a Department of Biochemistry and Biophysics, Texas A&M University, College Station, Texas 77843, United States; b Department of Biomedical Engineering, Texas A&M University, College Station, Texas 77843, United States; c Interdisciplinary Program in Genetics and Genomics, Texas A&M University, College Station, Texas 77843, United States; d Department of Biochemistry, 14716 University of Missouri, Columbia, Missouri 65211, United States; e Department of Ophthalmology, 14716 University of Missouri, Columbia, Missouri 65211, United States

## Abstract

Copper (Cu) is an essential trace element that serves as a cofactor for enzymes involved in diverse metabolic pathways and processes across multiple subcellular compartments. Mutations that cause systemic or subcellular Cu deficiency result in fatal pediatric disorders, including Menkes disease and related mitochondrial disorders. Treatment of these disorders requires pharmacological agents that can transport Cu across membranes; however, the only approved drug, Cu histidinate, shows limited efficacy. Using genetic models of Menkes disease, we recently demonstrated that the Cu ionophore elesclomol (ES) efficiently restores the activity of mitochondrial and cytosolic cuproenzymes, but its ability to metalate cuproenzymes within secretory compartments is low. To overcome this limitation, we investigated Cu nanoparticles (Cu-NPs) as a facile means of delivering Cu to multiple subcellular compartments simultaneously. Cu-NPs were synthesized by chemical reduction to produce spherical nanoparticles with an average size of 80 nm. Biophysical characterization of Cu-NPs revealed zero-valent Cu and a neutral zeta potential, compatible with biological interactions and in vivo circulation. In cellular models of Cu deficiency, Cu-NPs were shown to internalize via clathrin-mediated endocytosis and restore the activity of cuproenzymes in mitochondrial, cytosolic, and secretory compartments. Importantly, biomarkers of Cu toxicity were not activated under these conditions, indicating a favorable therapeutic index. Overall, these findings highlight Cu-NPs as versatile Cu delivery agents with therapeutic potential for Cu deficiency disorders.

## Graphical abstract

**Figure:**
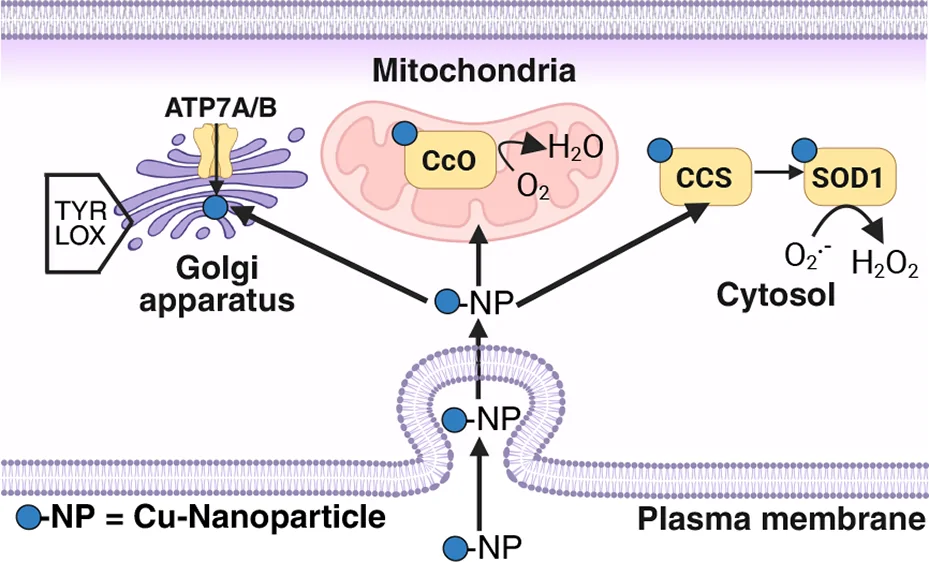

## Introduction

Copper (Cu) is an indispensable trace element that serves as a cofactor for several cuproenzymes involved in a wide range of cellular processes, including mitochondrial energy generation, pigment production, and the detoxification of reactive oxygen species.^1^ In eukaryotes, the high-affinity Cu permease, CTR1, mediates Cu uptake into the cytoplasm, where a network of chaperones, transporters, and small-molecule ligands is required to deliver Cu to three major sites of cuproenzyme synthesis: mitochondria, cytoplasm, and secretory pathway.^1^ In mitochondria, a diverse group of chaperones and assembly factors incorporate Cu into cytochrome *c* oxidase, the terminal enzyme of the electron transport chain. In the cytoplasm, the CCS chaperone delivers Cu to superoxide dismutase 1 (SOD1). However, the majority of cuproenzymes are either secreted from cells or localized to endomembrane compartments. Because such enzymes have lumenally oriented catalytic sites, their metalation requires Cu to be transported from the cytoplasm into the secretory pathway.^1^ In most cells, this critical process occurs via the Cu-transporting P-type ATPases ATP7A or ATP7B, which are located in the trans-Golgi network (TGN). Under excess Cu conditions, ATP7A and ATP7B traffic from the TGN to post-Golgi vesicles and/or the plasma membrane to facilitate cellular Cu export.

Pathogenic mutations in some of these Cu transport proteins give rise to fatal metabolic diseases.^2–7^ For example, loss-of-function mutations in ATP7A cause Menkes Disease (MD),^2^ a disorder of systemic Cu deficiency that is typically fatal by age three. MD is characterized by kinky hair, lax skin, hypotonia, seizures, and progressive neurodegeneration resulting in developmental delays and intellectual disability.^2^ More recently, mutations in the CTR1 were shown to cause a lethal pediatric disease with Menkes-like symptoms, including seizures, truncal hypotonia, and severe neuroatrophy.^8,9^ These genetic diseases underscore the indispensable role of Cu transport pathways in human physiology and highlight the urgent need for therapeutic strategies that deliver Cu to nascent cuproenzymes during biosynthesis in different subcellular compartments.

Daily subcutaneous injections of Cu-histidinate have been used to treat children with MD,^10–12^ and recently the Food and Drug Administration (FDA) approved Cu-histidinate for MD.^13^ While parenteral delivery of Cu-histidinate bypasses the ATP7A-dependent block in intestinal Cu absorption, its therapeutic efficacy is mostly seen in patients treated very early in life (<1 month of age) with mutations that retain partial ATP7A function.^12^ Indeed, only about half of patients treated early with Cu-histidinate are reported to survive beyond six years of age.^13^ The limited therapeutic efficacy of Cu-histidinate is thought to be due to inefficient delivery of Cu into the central nervous system, which requires ATP7A-dependent transport across the choroid plexus-epithelial barrier.^14^

Recently, our lab discovered the therapeutic potential of the Cu ionophore elesclomol (ES) for treating Cu deficiency disorders.^15–17^ ES binds Cu(II) and forms a hydrophobic, electrically neutral, square planar molecule that can transport Cu across biological membranes. Studies with cellular and animal models of Cu deficiency have shown that ES can efficiently deliver Cu to the mitochondrial and cytosolic cuproenzymes;^15,16^ however, its ability to deliver Cu to the Golgi and secretory pathway cuproenzymes is dependent on functional ATP7A, which pumps cytosolic Cu into the lumen of these organelles.^18^ Consistent with its mechanism of action, ES-Cu treatment of patients with MD showed remarkable improvement in their neurodevelopment;^19^ however, connective tissue defects persisted, likely due to insufficient metalation of cuproenzymes in the secretory pathway.^19^ Thus, there is a need to identify molecules that can bypass ATP7A and transport Cu into the secretory pathway compartments, particularly in patients with null mutations in the *ATP7A* gene.

Nanomedicine provides an innovative solution to this challenge. Nanoparticles, defined as materials with dimensions in the nanometer range, have been shown to traverse cellular membranes via endocytic pathways and facilitate intracellular drug delivery through the secretory network.^20^ In this study, we synthesized Cu nanoparticles (Cu-NPs) to test whether they can make Cu bioavailable to cuproenzymes localized in different subcellular compartments in cells lacking Cu transporters. Our results demonstrated the ability of Cu-NPs to deliver Cu to different subcellular compartments and activate cuproenzymes even in cells lacking the ATP7A transporter.

## Results and Discussion

### Chemical Synthesis and Biophysical Characterization of Cu-NPs

The primary objective of this study was to develop nanoparticles with versatile Cu-delivery capacity. To this end, we synthesized Cu nanoparticles (Cu-NPs) using the salt reduction method.^21^ To decrease cytotoxicity and improve cytocompatibility, we used FDA-approved polyethylene glycol as a coating agent. Dynamic Light Scattering of the synthesized Cu-NPs revealed an average particle size of ∼80 nm (Figure 1A). The measured zeta potential of approximately –9 mV is consistent with effective PEG coating of the nanoparticles, which likely minimizes their aggregation and agglomeration (Figure 1B). This surface potential falls within a near-neutral range generally associated with improved biocompatibility and reduced clearance, which may support their prolonged circulation in biological systems.^22^ The crystallite properties and chemical composition of the synthesized nanoparticles were determined by X-ray diffraction and X-ray photoelectron spectroscopy (Figure 1C, D), which show the expected spectra for elemental Cu, along with minor peaks corresponding to oxidized Cu.^21,23^ A weak Cu_2_O signal was also detected, which could be attributed to the surface oxidation of synthesized Cu-NPs (Figure 1C). Transmission electron microscopy demonstrated that the Cu-NPs possess a predominantly spherical morphology (Figure 1E, F). Taken together, the biophysical characterization confirmed the structure and composition of the synthesized Cu-NPs.

**Figure 1:**
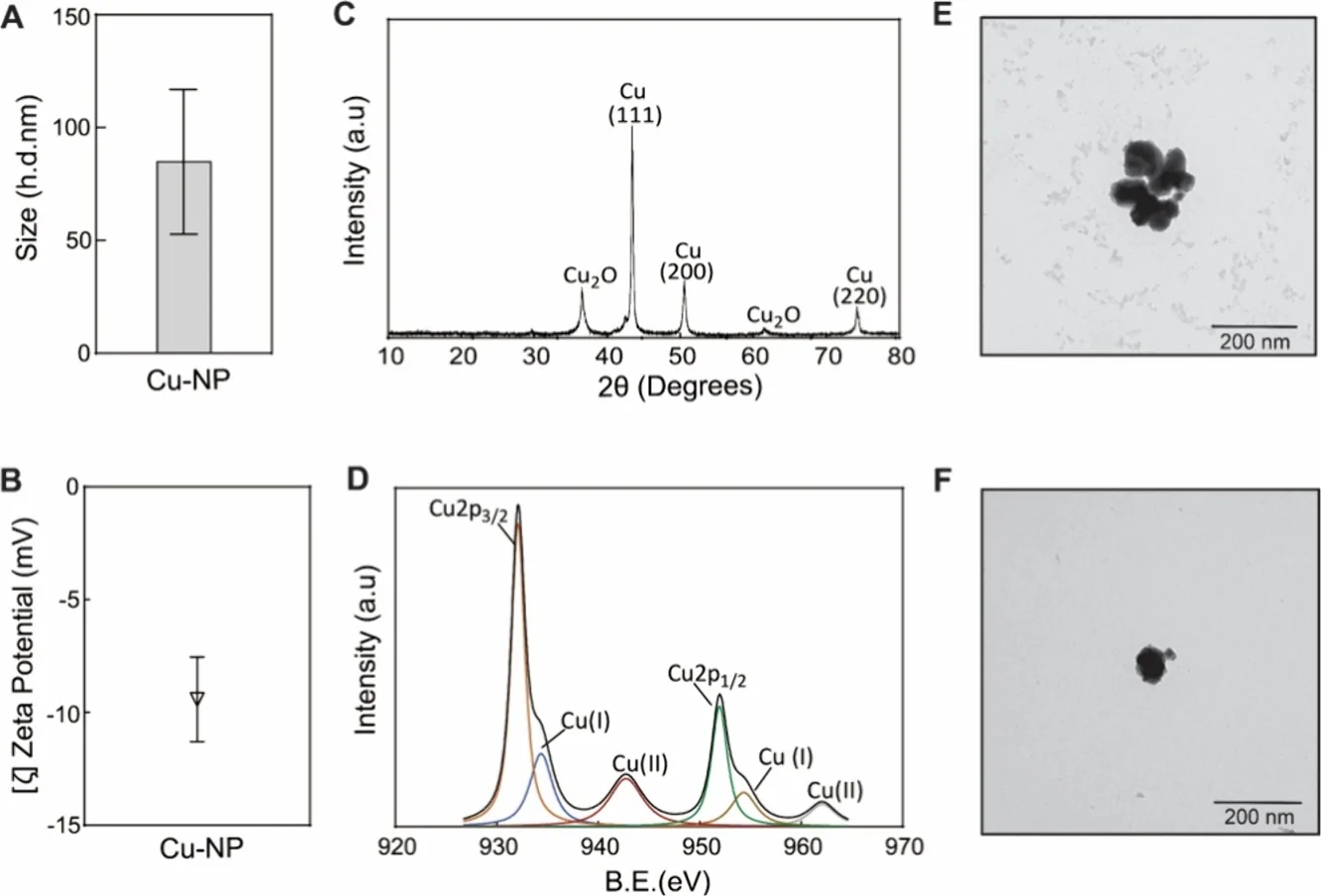
Biophysical Characterization of Cu-NPs. (A) Hydrodynamic diameter of Cu-NP. (B) Zeta-potential of Cu-NPs dissolved in ultrapure water. (C, D) XRD and XPS spectra of Cu-NPs. (E, F) TEM analysis of Cu-NPs suspended in water.

### Cellular Uptake of Copper Nanoparticles

To evaluate whether the synthesized Cu-NPs are bioactive and can deliver Cu to cells, we first determined their cell growth inhibitory concentration (IC_50_). To this end, we incubated wild-type (WT) and Cu-deficient (*Ctr1^–/–^*) H9c2 cardiomyocyte cells with increasing concentrations of Cu-NPs and measured their viability after 48 h of treatment. We found that the IC_50_ values for WT and *Ctr1^–/–^* H9c2 cells were 33 and 47 μg/mL, respectively (Figure 2A,B). To determine intracellular Cu accumulation, inductively coupled plasma-mass spectrometry (ICP-MS) was performed following Cu-NP treatment. Results indicate a significant increase in cellular Cu levels following Cu-NP supplementation (Figure 2C). Notably, Cu-NP supplementation also increased the intracellular Cu content of H9c2 *Ctr1^–/–^* cells, which lack the major cellular Cu transporter, CTR1, indicating that Cu-NPs can bypass CTR1-mediated Cu uptake (Figure 2C).

Previous studies have reported multiple endocytic routes of nanoparticle internalization into cells.^24^ To elucidate the cellular uptake route of Cu-NPs, we synthesized FITC-tagged Cu-NPs and measured their internalization in the presence of inhibitors of endocytosis. To distinguish between different endocytic pathways responsible for Cu-NP internalization, we used various inhibitors, including chlorpromazine hydrochloride to inhibit clathrin-mediated endocytosis, nystatin to inhibit caveolar-mediated endocytosis, and wortmannin to inhibit macropinocytosis and phagocytosis.^25^ Our results suggest that at the lower concentration of Cu-NP (4 μg/mL), the major uptake pathway involved is clathrin-mediated endocytosis (Figure 2D). At higher concentrations (20 μg/mL), the other endocytic pathways are also activated, although clathrin-mediated endocytosis remains the primary internalization pathway (Figure 2E). To further confirm that Cu-NPs are taken up by an endocytic route, we performed confocal microscopy to visualize the presence of Cu-NPs in various regions of the secretory pathway (early endosomes, Rab5; late endosomes, Rab7; lysosomes, LAMP1; and Golgi body, anti-58K) and the mitochondria (Figure S1). Our results showed that the Cu-NPs were internalized, but due to their low intensity, it was difficult to convincingly determine their co-localization within the subcellular compartments examined (Figure S1).

**Figure 2:**
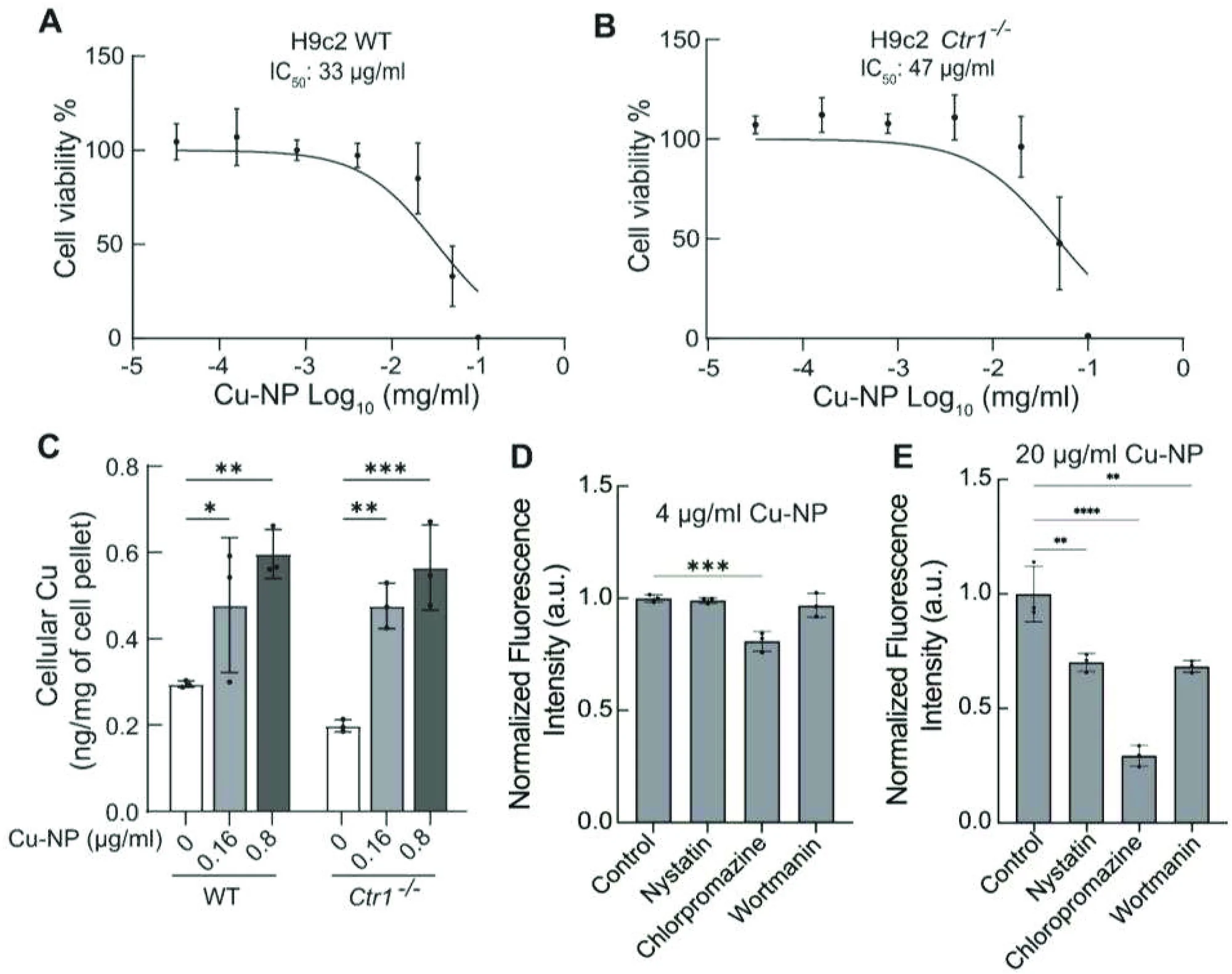
Intracellular accumulation and uptake of Cu-NPs. (A) IC_50_ of Cu-NPs for WT (A) and *Ctr1^*^–/–^*^* (B) H9c2 cells. Cells were treated with increasing concentrations of Cu-NPs and cell viability was measured using CellTitre-Glo after 48 h treatment. Each data point represents four biological and six technical replicates and is expressed as mean ± S.D., *n* = 4. (C) ICP-MS-based measurement of Cu levels in WT and *Ctr1^*^–/–^*^* H9c2 cells treated with the indicated concentrations of Cu-NPs, *n* = 3. (D) Flow cytometry analysis of *Ctr1^*^–/–^*^* H9c2 cells treated with FITC-tagged 4 μg/mL or (E) 20 μg/mL Cu-NPs in the presence of indicated inhibitors of endocytosis, *n* = 3. For statistical significance, we have used the log (inhibitor) vs normalized response for panels A and B, and one-way ANOVA for panels C, D, and E, respectively. ***P < 0.001, **P < 0.01, *P < 0.05.

Collectively, these findings indicate that the synthesized Cu-NPs are capable of cellular Cu delivery and can circumvent CTR1 dependency, prompting further investigation into their ability to restore Cu-dependent enzymatic functions in different subcellular compartments.

### Cu-NPs Mediate Cu Delivery to Secretory Compartment-Localized Cuproenzymes

Intracellular Cu does not exist in a free form; once Cu(I) enters the cytoplasm via the high-affinity transporter CTR1, it is rapidly bound by chaperones such as ATOX1, which delivers Cu to the ATP7A Cu transporter located in the trans-Golgi network.^1^ ATP7A-mediated Cu transport into the Golgi lumen facilitates metalation of nascent cuproenzymes en route through the secretory pathway, such as tyrosinase, lysyl oxidase, and dopamine-β-hydroxylase.^1^ To assess whether Cu released from Cu-NPs is available to secretory compartment-localized cuproenzymes, we utilized B16 murine melanoma cells as a cellular model of tyrosinase-mediated melanogenesis. These cells express tyrosinase in the specialized secretory pathway compartments called melanosomes, where it receives Cu via the ATP7A Cu pump localized on the melanosome membrane. Consistent with the known requirement for ATP7A-dependent Cu delivery to tyrosinase, cell pellets derived from B16 WT cells are visibly pigmented, whereas a derivative cell line lacking the ATP7A transporter (B16 *Atp7a^–/–^*) is amelanotic and produces white cell pellets (Figure S2).

**Figure 3:**
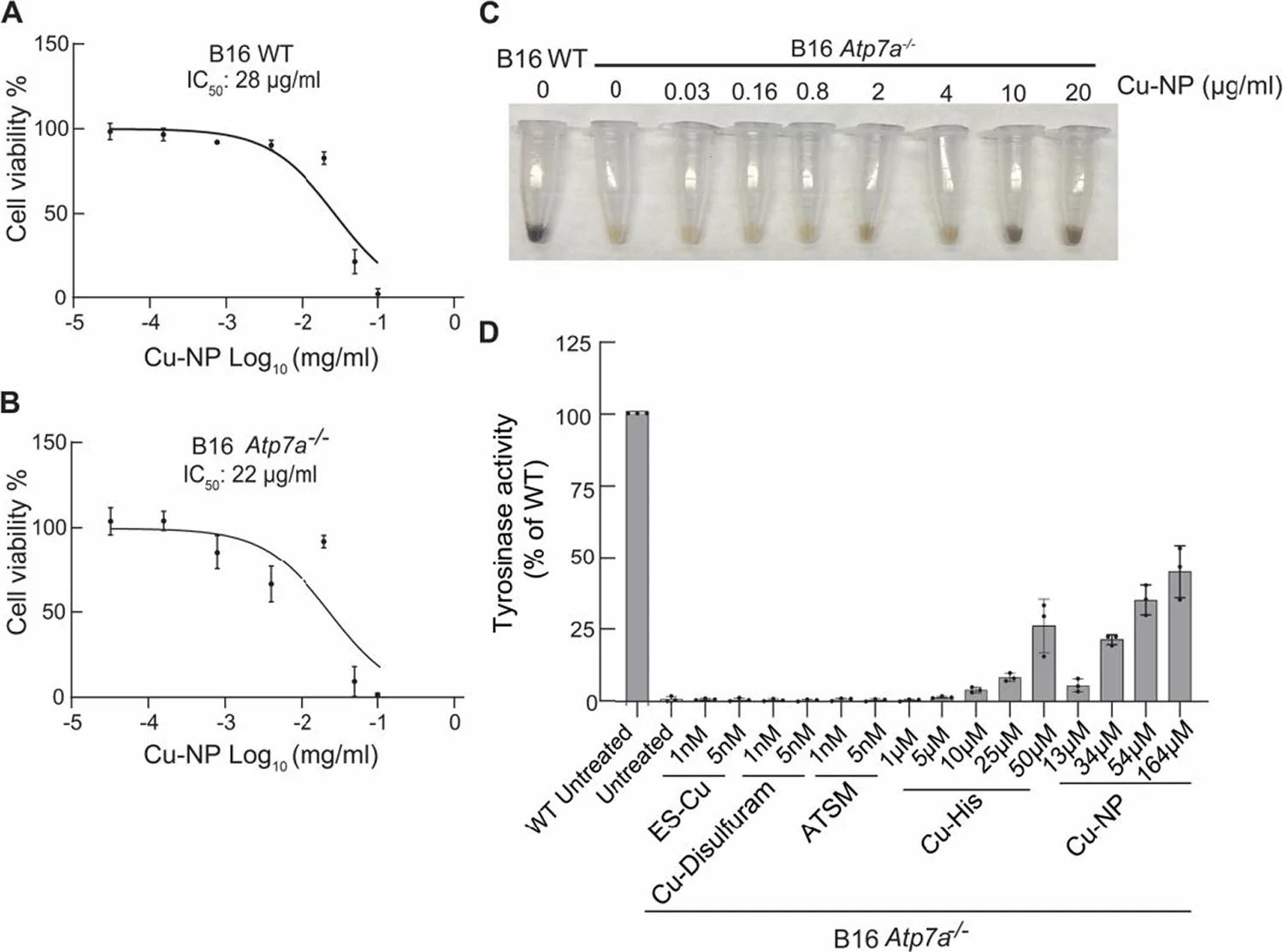
Cu-NPs bypass the loss of ATP7A-dependent metalation of tyrosinase. (A, B) IC_50_ of Cu-NPs for WT (A) and *Atp7a^*^–/–^*^* (B) B16 cells. Cells were treated with increasing concentrations of Cu-NPs, and cell viability was measured using CellTitre-Glo after 48 h treatment. Each data point represents three biological and six technical replicates and is expressed as mean ± S.D., *n* = 3. (C) Cell pellets of wild-type and *Atp7a^*^–/–^*^* B16 cells treated with indicated concentrations of Cu-NPs. (D) Tyrosinase activity measured in wild-type and *Atp7a^*^–/–^*^* B16 cells treated with different drugs (indicated concentrations), *n* = 3. For statistical significance, we have used the log (inhibitor) vs normalized response for panels A and B.

To test the ability of Cu-NPs in making Cu bioavailable to tyrosinase, we first determined the maximal tolerated concentrations of Cu-NPs by measuring the viability of B16 melanoma cells following a two-day treatment with increasing doses of Cu-NPs. Our results indicate that the IC_50_ of Cu-NPs for WT and *Atp7a^–/–^* B16 melanoma cells is 28 and 22 μg/mL, respectively (Figure 3A, B). Notably, these IC_50_ values are lower than those of Cu salts, such as Cu-histidinate and CuCl_2_, suggesting that the mechanism of Cu uptake via Cu-NPs is distinct from those of these Cu salts (Figure S3). A possible explanation for the difference in IC_50_ values for Cu-NPs vs Cu salts such as CuCl_2_ is that the uptake of Cu ions from Cu salts occurs via plasma membrane transporters such as CTR1, which allow regulated entry of Cu ions. Cells have evolved multiple homeostatic mechanisms to defend against this physiological entry route, including Cu-induced endocytosis and degradation of CTR1,^26^ as well as induction of metallothioneins that sequester excess cytosolic Cu.^1^ In contrast, Cu-NPs are internalized predominantly by endocytic pathways, resulting in the accumulation of Cu within endolysosomal compartments. Because this mode of Cu entry bypasses the tightly regulated plasma membrane uptake machinery, the resulting intracellular Cu burden may be less effectively controlled by canonical Cu homeostatic mechanisms. Consequently, Cu released from endolysosomal stores may produce greater cellular toxicity than an equivalent amount of Cu delivered through transporter-mediated uptake, thereby accounting for the increased susceptibility of cells to Cu-NPs relative to soluble Cu salts.

Next, we tested whether Cu-NPs at subtoxic doses can bypass the loss of ATP7A-dependent tyrosinase activity. B16 Atp7a^*–/–*^ cells exposed to increasing concentrations of Cu-NPs restored pigmentation in a dose-dependent manner (Figure 3C). To complement this qualitative readout of restoration of tyrosinase activity, we performed quantitative measurement of tyrosinase enzymatic activity in B16 *Atp7a^–/–^* cell lysates, revealing a dose-dependent increase in activity from 8 to 47% of B16 WT levels (Figure 3D). Notably, the ability of Cu-NPs to activate tyrosinase was superior to Cu-histidinate, whereas other Cu-transporting drugs, including elesclomol-Cu, Cu-Disulfiram, and Cu-ATSM, did not rescue tyrosinase activity at their non-toxic doses (Figure 3D).

**Figure 4:**
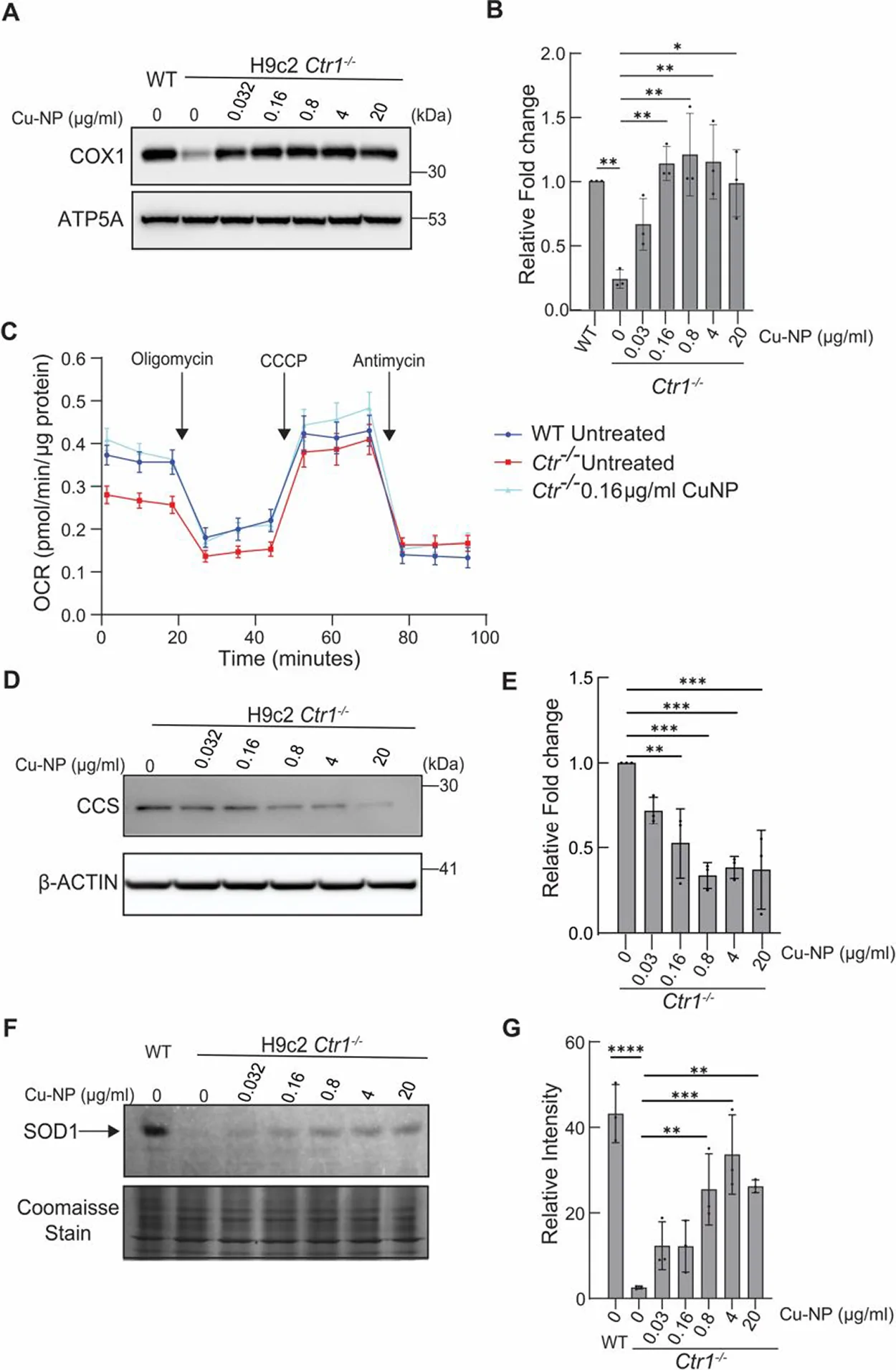
Cu from Cu-NPs is bioavailable to mitochondrial and cytosolic cuproenzymes. (A, B) Immunoblotting and quantification of cytochrome *c* oxidase subunit I (COX1) in whole-cell lysates from WT and *Ctr1*^–/–^** H9c2 cells treated with the indicated concentrations of Cu-NPs for 48 h. ATP5A is used as a loading control. For quantification, band intensities of COX1 were normalized to the corresponding ATP5A, *n* = 3. (C) Oxygen consumption rate (OCR) measured in WT and *Ctr1^*^–/–^*^* H9c2 cells (untreated and treated with 0.16 μg/mL Cu-NP). ATP-coupled, maximal, and non-mitochondrial respiration, were measured by using oligomycin, CCCP, and antimycin A, respectively. Data are shown as mean ± SEM, *n* = 3. (D, E) *Ctr1^*^–/–^*^* H9c2 cells were treated with indicated concentrations of Cu-NPs and cytosolic cuproprotein, CCS abundance was determined using western blotting. β-Actin was used as loading control and for normalization, *n* = 3. (F) In-gel SOD1 activity in WT and *Ctr1^*^–/–^*^* H9c2 cells treated with indicated concentrations of Cu-NPs for 48 h. Coomassie stain of the same gel is used as loading control. A representative image from three independent experiments is shown. (G) Quantification of data from panel (F), *n* = 3. For statistical significance, we have used one-way ANOVA for panels B, E, and G respectively. ***P < 0.001, **P < 0.01, *P < 0.05.

### Cu-NPs Make Cu Bioavailable to Mitochondrial and Cytosolic Cuproenzymes

To determine if the Cu delivered by Cu-NPs is also accessible to other subcellular organelles, such as mitochondria, we tested the ability of Cu-NPs to increase the abundance of the mitochondrial cytochrome *c* oxidase subunit COX1 in cells lacking the Cu importer CTR1. Since the stability of COX1 depends upon Cu coordination, its abundance serves as a reliable marker of mitochondrial Cu status.^15^ Unlike compartment-specific Cu deficiency, loss of plasma membrane-localized Cu transporter CTR1 is expected to decrease whole-cell Cu content, including that of mitochondria. Therefore, we used *Ctr1^–/–^* H9c2 rat cardiomyocytes, which are rich in mitochondria. We incubated WT and Cu-deficient *Ctr1^–/–^* H9c2 cardiomyocytes with non-toxic concentrations of Cu-NPs and probed for the abundance of COX1 by western blotting. As expected, *Ctr1^–/–^* cells showed markedly reduced abundance of COX1 as compared to WT, reflecting Cu deficiency in mitochondria (Figure 4A, B). Notably, Cu-NP supplementation restored COX1 levels in *Ctr1^–/–^* cells to those of WT, suggesting that Cu derived from Cu-NP is accessible to mitochondrial cuproproteins (Figure 4A, B). To test whether the restored COX1 results in increased mitochondrial respiration, we measured oxygen consumption rate (OCR) of WT and *Ctr1^–/–^* cells using an XF24 extracellular flux analyzer and found a complete rescue of respiration upon treatment with 0.16 μg/mL Cu-NP (Figure 4C). Notably, Cu-NPs restored all aspects of respiration in *Ctr1^–/–^* cells to WT levels, including basal-, ATP-coupled (upon oligomycin treatment), and maximal (upon CCCP treatment) respiration rates (Figure 4C).

Next, to assess if the Cu-NPs can deliver Cu to cytosolic cuproenzymes, we examined the Cu chaperone for superoxide dismutase (CCS) as a proxy for cytosolic Cu bioavailability. CCS delivers Cu to superoxide dismutase 1 (SOD1), and its abundance inversely correlates with the bioavailable Cu pool in the cytosol. Cu-NP treatment of *Ctr1^–/–^* cells reduced CCS abundance in a dose-dependent manner (Figure 4D,E), suggesting that Cu-NPs effectively increase cytosolic Cu availability. To determine if the decrease in CCS abundance is due to cytosolic Cu availability and not due to non-specific effects of Cu-NPs treatment on protein turnover or oxidative stress, we measured SOD1 activity. Consistent with Cu-dependent changes in CCS abundance, our results indicate that Cu-NPs stimulated SOD1 activity in a dose-dependent manner (Figure 4F, G).

### Cu-NPs Do Not Induce Cuproptosis at Their Efficacious Concentrations

A potential risk of excess Cu is cuproptosis,^27^ a recently described form of cell death. Cuproptosis is characterized by disruption of Fe–S clusters, accompanied by decreased abundance of Fe–S-cluster-containing proteins, a decrease in abundance of lipoylated proteins, and appearance of higher-order oligomers of lipoylated proteins^27^ (Figure 5A). To determine the concentration(s) at which Cu-NPs trigger cuproptosis, we measured the abundance of representative Fe–S-cluster-containing proteins, including aconitase 2 (ACO2), lipoic acid synthetase (LIAS), succinate dehydrogenase subunit B (SDHB), and ubiquinol-cytochrome *c* reductase Rieske iron-sulfur polypeptide 1 (UQCRFS1) upon treatment with varying concentrations of Cu-NPs. Our results show that only SDHB abundance is reduced when cells are exposed to 20 μg/mL or higher concentrations of Cu-NPs (Figure 5B).

**Figure 5:**
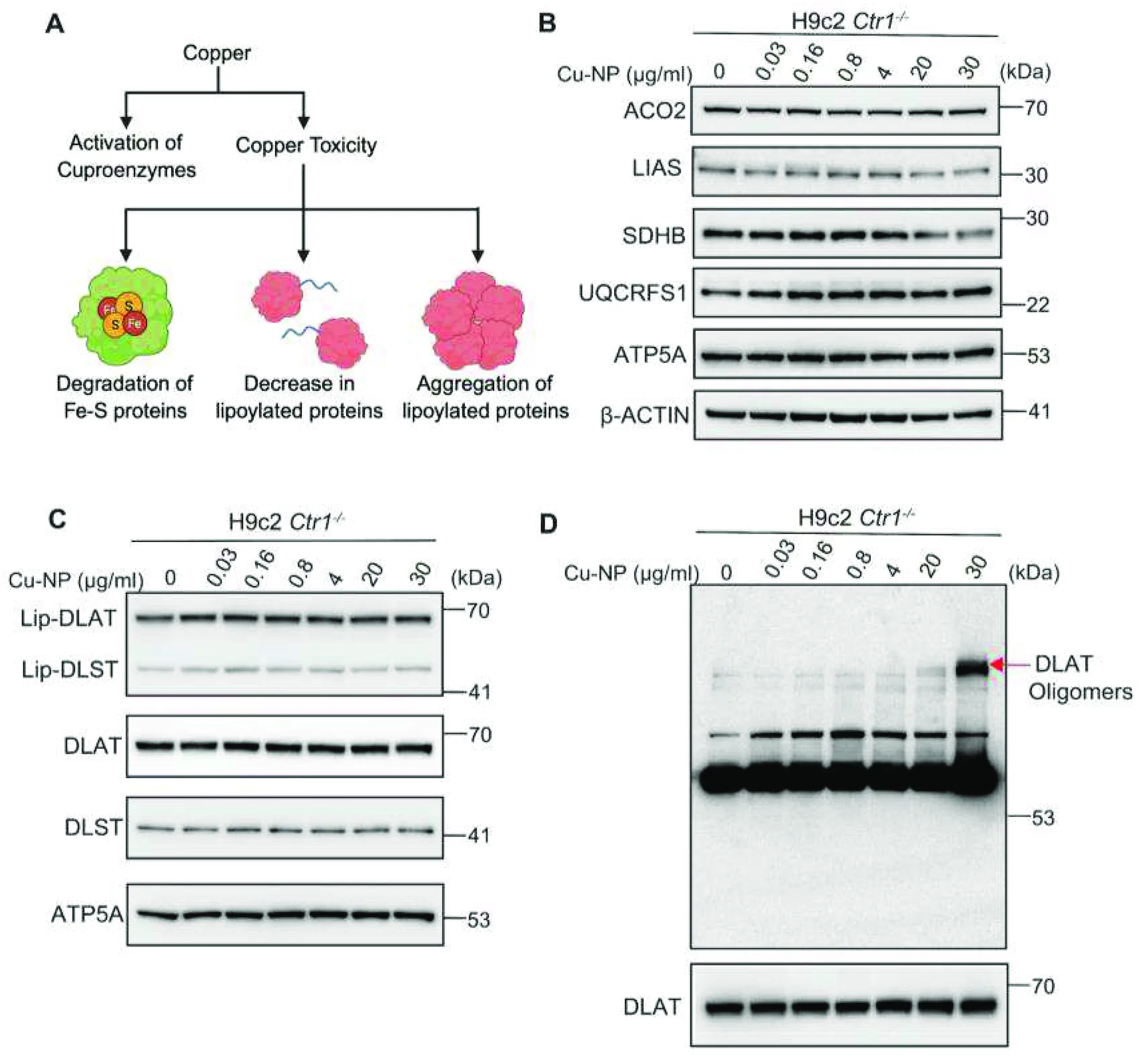
Efficacious dose of Cu-NPs does not elevate biomarkers of cuproptosis. (A) Schematic depiction of Cu-mediated cytotoxicity. (B, C) Western blot analysis of iron–sulfur (Fe–S) cluster (B), lipoic acid containing proteins (C). (D) DLAT oligomers in whole cell lysates of *Ctr1^^–/–^^* H9c2 cells after treatment with indicated concentrations of Cu-NPs. ATP5A and β-Actin were used as a loading control, *n* = 3.

We next measured the abundance of lipoylated proteins following treatment with increasing concentrations of Cu-NPs. To this end, we focused on the two key mitochondrial enzymes that regulate carbon entry into the tricarboxylic acid cycle—dihydrolipoamide *S*-acetyltransferase (DLAT) and dihydrolipoamide succinyltransferase (DLST)—which are lipoylated by LIAS. Our results showed that their levels remained unchanged even at higher concentrations of Cu-NPs (20–30 μg/mL), indicating preserved lipoylation status (Figure 5C). To evaluate aggregation of lipoylated proteins—a hallmark of cuproptosis^27^—we performed non-reducing gel electrophoresis to detect DLAT oligomerization following treatment of cells with increasing concentrations of Cu-NPs. Higher-molecular-mass oligomers (approximately 90–150 kDa) were observed only at 30 μg/mL Cu-NP supplementation (Figure 5D). Taken together, our results demonstrate that Cu-NPs do not induce cuproptosis at concentrations that bypass the loss of CTR1 and ATP7A Cu transporters, thereby supporting further evaluation as Cu delivery agents in vivo.

## Conclusions

Cu deficiency disorders, such as Menkes disease, are caused by impaired Cu transport and subsequent loss of cuproenzyme activity in different subcellular compartments.^1^ Treatment with recently approved Cu-histidinate has had limited success in ameliorating disease pathology.^12,13^ Thus, new Cu delivery strategies are needed.

**Figure 6:**
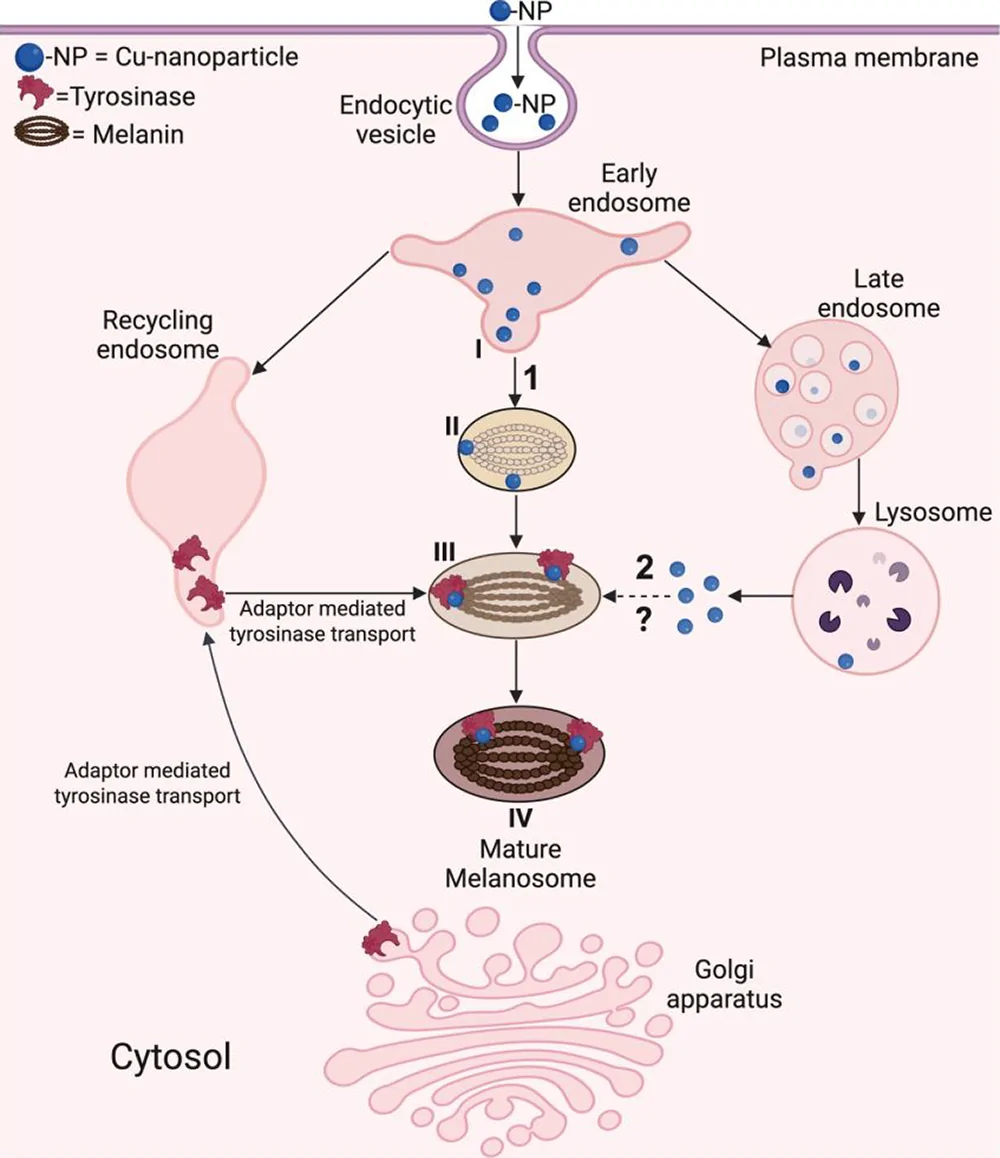
Model describing cellular uptake and intracellular trafficking of Cu-NPs to secretory compartments. Cu-NPs, internalized by endocytosis, first reach early endosomes, from where they can follow two main non-mutually exclusive paths. (1) Cu-NPs are sorted into stage I premelanosomes, and when they mature into stage III melanosomes and receive apo-tyrosinase, Cu derived from Cu-NPs becomes available for tyrosinase metalation. (2) Cu-NPs are routed to late endosomes and then to lysosomes, where local dissolution of Cu-NPs releases Cu. This Cu is exported from lysosomes into the cytosol, feeds into the cellular Cu network, and a portion is subsequently transported to stage III melanosomes via unknown transport processes, where it metalates apo-tyrosinase to generate active holoenzyme.

In this study, we synthesized and characterized PEG-coated Cu-NPs as a potential Cu delivery platform. PEG coating renders Cu-NPs more hydrophilic, which may improve circulation time and reduce immune recognition.^22^ PEGylation, while beneficial in improving compatibility and providing a modular surface for introducing targeting ligands, has additional implications on intracellular trafficking.^28^ The subcellular localization of nanoparticles is heavily influenced by their morphology, size, shape, and charge. PEGylation of Cu-NPs aids in their internalization, retention, and intracellular localization within the cell.^28,29^ Importantly, Cu-NPs enter cells through endocytic pathways that are independent of high-affinity Cu transporter CTR1, enabling cellular uptake even when canonical Cu transport pathways are disrupted.

Our results demonstrate that Cu delivered by Cu-NPs becomes bioavailable to cuproproteins localized in multiple intracellular compartments, including the secretory compartments, mitochondria, and cytosol. Our study raises an important question as to why Cu-NPs behave differently from Cu salts or small-molecule Cu complexes. The answer probably lies in the route of internalization—they are internalized predominantly via receptor-mediated endocytosis, unlike ionic Cu or small-molecule Cu complexes, which are either internalized through transporter(s) or via passive diffusion through the membrane (i.e., ionophores). This point is critical for understanding the rescue of the secretory pathway cuproenzyme tyrosinase by Cu-NPs in melanocytes. Previous studies have shown that melanosomes mature through four distinct stages: stages I and II are premelanosomes lacking pigment, whereas stage III melanosomes acquire tyrosinase, and stage IV is fully melanized^30,31^ (Figure 6). Tyrosinase is sorted from endosomes to stage III melanosomes via clathrin-dependent carriers, which function independently to route tyrosinase from the trans-Golgi network and early/recycling endosomes. Notably, tyrosinase acquires Cu only transiently and inefficiently within the trans-Golgi network of melanocytes; instead, full loading with Cu occurs in maturing melanosomes, where ATP7A delivers Cu to the melanosomal lumen.^32^ In this context, our finding that Cu-NP treatment in ATP7A-knockout cells activates tyrosinase and restores melanin synthesis indicates that Cu can reach stage III melanosomes via an ATP7A-independent route. Based on this information, we propose two non-mutually exclusive mechanisms for Cu delivery to tyrosinase in secretory vesicles (Figure 6). The first path involves direct routing of Cu-NPs into the melanosome lineage through the early endosome, and the second path involves lysosomal processing of endocytosed Cu-NPs and subsequent delivery to melanosomes (Figure 6).

The broad subcellular accessibility distinguishes Cu-NPs from several existing Cu delivery approaches that often exhibit limited compartment-specific activity. Furthermore, Cu-NPs did not induce detectable signatures of cuproptosis at concentrations that restored cuproenzyme function, suggesting a favorable safety profile under the conditions tested. Taken together, these findings establish Cu-NPs as a versatile Cu delivery modality with potential applications in both basic research and the development of therapeutic strategies for disorders of Cu deficiency.

## Methods

### Chemicals

CuSO_4_ (anhydrous, 98%), polyethylene glycol (PEG) M.W. 6000, and hydrazine monohydrate (98.0%) were purchased from VWR and Sigma. Type 1 ultrapure water used was obtained using a Barnstead E-Pure ultrapure water system (Thermo Scientific). Elesclomol-Cu was purchased from Accel Pharmtech; disulfiram, Cu-ATSM, carbonyl cyanide 3-cholorophenylhydrazone (CCCP), oligomycin, and antimycin A were purchased from Sigma.

### Cu-NP Synthesis

Cu-NPs were synthesized via a chemical reduction method to obtain metallic Cu nanoparticles following previously reported protocols with minor modifications. The reaction involves mixing Cu salt and hydrazine as a reducing agent in an aqueous PEG solution. The initial concentrations of the reaction components were 0.015 mol/L for CuSO_4_ and 0.04 mol/L for PEG. Precursors were dissolved in Type 1 ultrapure water. Hydrazine monohydrate was added to the reaction mixture at 3% v/v in one step with constant stirring at 300 rpm at room temperature. Reduction was monitored by a color change, from light blue to dark blue (after 1 min) to Brown (after 5 min) and finally amber (after 25 min). The resulting final product is washed with increasing concentrations of EtOH (0, 25, 50, 75, 100%) and resuspended in ultrapure water for subsequent freezing and lyophilization. The final powder is stored under vacuum.

### Cu-NP Characterization

Nanoparticle physical properties such as size, charge, and shape were analyzed via Dynamic light scattering (DLS), Zeta Potential (Zetasizer Nano Series (Malvern Instruments)), and transmission electron microscopy (TEM). TEM was performed on a carbon grid at an accelerating voltage of 200 kV using a JEOL-JEM 1200 transmission electron microscope. For sample preparation, aqueous dispersions of Cu-NP samples were drop-casted and air-dried on a Cu grid (Ted Pella Inc.). The crystallographic phase of Cu-NP was confirmed by X-ray powder diffraction (Bruker D8 Advanced). Briefly, the sample was placed in the sample holder of a two-circle goniometer, enclosed in a radiation safety enclosure. The X-ray source was a 2.2 kW Cu X-ray tube, maintained at an operating current of 40 mA at 40 kV. The X-ray optics was the standard Bragg-Brentano para-focusing mode with the X-ray diverging from a DS slit (1 mm) at the tube to strike the sample and then converging at a position-sensitive X-ray Detector (Lynx-Eye, Bruker-AXS). The two-circle 250 mm diameter goniometer was computer controlled with independent stepper motors and optical encoders for the θ and 2θ circles with the smallest angular step size of 0.0001°2θ. Data collection was performed by the automated COMMANDER program by employing a DQL file. Data were analyzed by the program EVA, and the obtained peaks were indexed using JCPDS for Cu (4105040). The crystallographic arrangement of Cu atoms within the nanoparticles was determined using X-ray photoelectron spectroscopy (XPS, Omicron Inc.). The binding energy (B.E.) for Cu (2p) was recorded. The raw data were further processed and deconvoluted by XPS-41 multiple peak fit software and indexed using the standard library.

### Mammalian Cell Culture

The cell lines used in this study were rat H9c2 cardiomyocytes and murine B16 melanocytes. H9c2 WT and *Ctr1^–/–^* cells were grown in high-glucose Dulbecco's Modified Eagle Medium (DMEM) supplemented with 10% fetal bovine serum (FBS) (Sigma). For B16 WT and *Atp7a^–/–^* knockout cells, DMEM was supplemented with 10% FBS (Sigma), 2 mM glutamine, and 100 units/mL pen-strep (Life Technologies, Carlsbad, CA). Cells were cultured under 5% CO_2_ at 37 °C.

### Cell Viability Assay

Cells were plated at a density of 5000 cells/well in 96-well plates in their respective growth media and incubated at 37 °C in a CO_2_ incubator with a humidified atmosphere containing 5% CO_2_. After 12–16 h, the media in each well were replaced with fresh growth medium containing different concentrations of Cu-NPs and other specified drugs (refer to Table S1 for Cu-NP concentration in μM). Cells were further incubated for 48 h, followed by determination of cell viability using the CellTiter-Glo Cell Viability Assay (Promega).

### Sodium Dodecyl Sulfate-Polyacrylamide Gel Electrophoresis (SDS-PAGE) and Immunoblotting

Cells were plated at a seeding density of 4.0 × 10^5^ cells in a 100 mm dish and incubated at 37 °C in a CO_2_ incubator. After 12–16 h, the spent media was replaced with fresh growth media containing different concentrations of Cu-NPs, followed by further incubation for 48 h. The cells were then washed and harvested using cold phosphate-buffered saline (PBS). For sample preparation, the cell pellet was lysed on ice in radioimmunoprecipitation assay (RIPA) extraction buffer (Boston BioProducts, BP-115), supplemented with a 1× complete protease inhibitor cocktail (Roche), for 20 min, followed by centrifugation at 20,000 × *g* for 10 min at 4 °C. The supernatant was collected, and protein concentration was measured using the bicinchoninic acid (BCA) assay (Pierce). Equal amounts of protein samples (30 μg) were then loaded onto 12% NuPAGE Bis–Tris gel (Thermo Fisher Scientific) followed by protein fractionation and immunoblotting using the following primary antibodies: COX1, 1:1000 (ab14705; Abcam); CCS, 1:1000 (sc-55561; Santacruz), and β-Actin,1:100,000 (A2228, Sigma); ACO2, 1:1000 (MA535527; Thermo Fisher Scientific and 67509-1-Ig; Proteintech); LIAS, 1:1000 (115771AP; Proteintech); SDHB, 1:1000 (ab14714; Abcam); UQCRFS1, 1:1000 (ab14746; Abcam); DLAT, 1:1000 (PA56007; Thermo Fisher Scientific); DLST, 1:1000 (Thermo Fisher Scientific), ATP5A, 1:20,000 (ab14748; Abcam), and anti-Lipoyl antibody, 1:1000 (437695; Sigma and ab58724; abcam). After overnight incubation at 4 °C with primary antibodies, the blots were washed five times with 20 mM Tris-buffered saline containing 0.1% Tween 20 (TBST), incubated at room temperature with horseradish peroxidase-conjugated anti-rabbit (32460; Thermo Fisher Scientific) or anti-mouse (95017-332; VWR) secondary antibodies. After one hour, the blots were washed again with TBST (5×) and developed using Clarity or Clarity Max Western ECL Substrate (Bio-Rad).

### DLAT Oligomerization

RIPA extracts from H9c2 *Ctr1^–/–^* cells treated with indicated concentrations of Cu-NPs were used to determine DLAT oligomerization. Samples were prepared by adding 1× NuPAGE LDS Sample Buffer (Thermo Fisher Scientific) to 30 μg of protein RIPA extract. Proteins were fractionated under non-reducing conditions in 4–12% NuPAGE Bis–Tris gel (Thermo Fisher Scientific) followed by immunoblotting using an anti-DLAT antibody.

### Oxygen Consumption Rate (OCR) Measurements

H9c2 WT and *Ctr1^–/–^* cells were seeded at a density of 5000 cells/well in XF24-well cell culture microplates (Agilent Technologies) in 250 μL of standard growth media and incubated at 37 °C in a 5% CO_2_ incubator. After 12–14 h, the media in each well was replaced with fresh media (500 μL) containing indicated concentrations of Cu-NPs, followed by 48 h incubation at 37 °C. Before measurements, the media was replaced with 775 μL of fresh growth media, and cells were incubated at 37 °C for 30 min. Basal respiration measurements were carried out in intact cells using the Seahorse XF24 Extracellular Flux Analyzer (Agilent Technologies). For the mitochondrial stress test, oligomycin, carbonyl cyanide 3-cholorophenylhydrazone (CCCP), and antimycin A were prepared in DMEM without serum and sequentially added to final concentrations of 1, 25, and 1 μM, respectively. Following the assay, cells were washed three times with 500 μL PBS, and protein was extracted using 25 μL RIPA lysis buffer. Protein concentrations were determined using the BCA assay.

### Tyrosinase Activity

B16 *Atp7a^–/–^* cells were treated with specified concentrations of Cu-NPs. For qualitative analysis, visual change in cell pellet color was determined after 5 days of Cu-NP treatment. For quantitative analysis, the tyrosinase activity was measured using a colorimetric assay with minor modifications.^33^ Briefly, cells were incubated in Cu-NP-supplemented media for 72 h, followed by washing with cold PBS before harvesting by centrifugation. The cell pellet was then lysed using RIPA buffer containing 1× complete protease inhibitor cocktail (Roche) for 15 min on ice with gentle agitation followed by centrifugation at 15,000 × *g* for 30 min at 4 °C. Protein concentrations were measured using BCA assay and tyrosinase activity was measured (absorbance at 475 nm) in a clear 96-well plate in triplicate by incubating 40 μg of protein lysate with reaction mixture containing 50 mM phosphate buffer (pH 6.8) and 0.05% L-DOPA.

### Flow Cytometry

*Ctr1^–/–^* cells were seeded in a 6-well plate for 12–16 h in a humidified incubator at 37 °C containing 5% CO_2_. The spent media was replaced with fresh media containing different inhibitors of endocytic pathways, including chlorpromazine hydrochloride (inhibits clathrin-mediated endocytosis), nystatin (inhibits caveolar-mediated endocytosis), and wortmannin (inhibits micropinocytosis and phagocytosis). After 30 min, the cells were washed with PBS and treated with fresh media supplemented with FITC-tagged Cu-NPs for 4 h. Cu-NP uptake was then measured using the BD Accuri C6 flow cytometer after trypsinization and washing the cells with PBS.

### Inductively Coupled Plasma Mass Spectrometry (ICP-MS)

WT and *Ctr1^–/–^* H9c2 cells were treated with Cu-NPs for 48 h and harvested in cold PBS. For ICP-MS sample preparation, cell pellets were washed twice with 1 mL of 0.9% NaCl containing 100 μM ethylenediamine tetraacetic acid (EDTA) prepared in ultrapure metal-free water (Trace SELECT; Sigma), followed by two more washes with 0.9% NaCl to eliminate EDTA. Samples were then digested with 40% (w/v) nitric acid (Trace SELECT; Sigma) at 90 °C for 18 h, followed by 6 h digestion with 0.75% H_2_O_2_ (Sigma Supelco). The final sample volume was made to 6 mL with ultrapure water to make the final concentration of HNO_3_ 1.5% before injecting into the Perkin Elmer NexION 300D instrument. The calibration curve was prepared by diluting the ICP-MS complete standard solution (BDH82026-108, VWR) in 1.5% HNO_3_.

### Confocal Microscopy

Imaging of *Ctr1^–/–^* H9c2 cells treated with Cu-NPs was performed using a confocal microscope (Leica Sp8). Cells were grown in chambered coverslips (Ibidi) followed by treatment with Cu-NPs for 4 h (endosomal and lysosomal staining) or 24 h (Golgi or mitochondrial staining). After Cu-NP treatment, cells were washed with PBS and fixed with 4% paraformaldehyde (PFA) for 15 min at RT, followed by permeabilization with 0.1% Triton X-100 for 10 min at room temperature. For Mitotracker, cells were fixed and permeabilized after staining with Mito Tracker (Thermo Fisher). Cu-NPs were imaged using reflective light. Nucleus and actin were visualized using DAPI (Biotium) and Rhodamine phalloidin (Biotium), respectively. For visualization of early endosomes, late endosomes, lysosomes, and the Golgi body, the following primary antibodies were used (Abcam: ab27043, ab28624; Thermo Fisher: PA5-52369, MA1-164), followed by incubation with secondary antibodies conjugated with Alexa Fluor.

### In Gel SOD1 Activity

The WT and *Ctr1^–/–^* H9c2 cells were treated with Cu-NPs for 48 h. The cells were then washed and harvested using cold phosphate-buffered saline (PBS). For sample preparation, the cell pellet was lysed in cell lysis buffer (20 mM potassium phosphate, pH 7.4, 1 mM EDTA, 0.5% Triton X-100, 4 mM PMSF with protease inhibitor cocktail) by incubating on ice for 15 min. The supernatant was collected after centrifugation at 20,000 × *g* for 15 min, followed by protein concentration measurement by BCA assay. Then, 20 μg of protein supplemented with NativePAGE sample buffer (4×) was separated using a 4–16% native PAGE gel. The gel was stained for SOD1 activity as described previously.^34^

## Supplementary Material



## Data Availability

All data supporting the findings in this article are available in the main text or the supplementary information.

## Supporting Information

Confocal microscopy, color of cell pellets, IC_50_ analysis for Cu-histidinate and CuCl_2_, and supplementary table for Cu content in Cu-NPs (PDF)
