# Supplementary material for: Copper Nanoparticles Enable Multi-Compartment Cuproenzyme Metalation in Cellular Models of Copper Deficiency
Source: ACS Chem Biol. 2026 Aug 13;21(9):2441–50. doi: 10.1021/acschembio.6c00564 (PMC13632923; doi:10.1021/acschembio.6c00564)
Supplement: Supplementary file 1 [file cb6c00564_si_001.pdf]

Supporting information for

Copper Nanoparticles Enable Multi-Compartment Cuproenzyme Metalation in Cellular Models of Copper Deficiency

Harman Kaur[a], John Soukar[b,c], Nikita Gudekar[d,e], Vinit C. Shanbhag[d,e], Michael J. Petris[d,e], Akhilesh K. Gaharwar[b,c], and Vishal M. Gohil[a,c*]

[a]Department of Biochemistry and Biophysics, Texas A&M University, College Station, TX 77843, USA
[b]Department of Biomedical Engineering, Texas A&M University, College Station, TX 77843, USA
[c]Interdisciplinary program in Genetics and Genomics, Texas A&M University, College Station, TX 77843, USA
[d]Department of Biochemistry, University of Missouri, Columbia, MO 65211, USA
[e]Department of Ophthalmology, University of Missouri, Columbia, MO 65211, USA

[*]Correspondence should be addressed to Vishal M. Gohil: vgohil@tamu.edu

This pdf inculdes:

Figure S1: Confocal microscopy of *Ctr1*$^{-/-}$ H9c2 cells treated with Cu-NPs........S2
Figure S2: Wild-type (WT) and *Atp7a*$^{-/-}$ B16 cell pellets........S3
Figure S3: IC_{50} of Cu-Histidinate and $CuCl_2$ for WT (A, C) and *Atp7a*$^{-/-}$........S4
Table S1: Concentrations of Cu-NPs (μM) measured using ICP-MS........S5

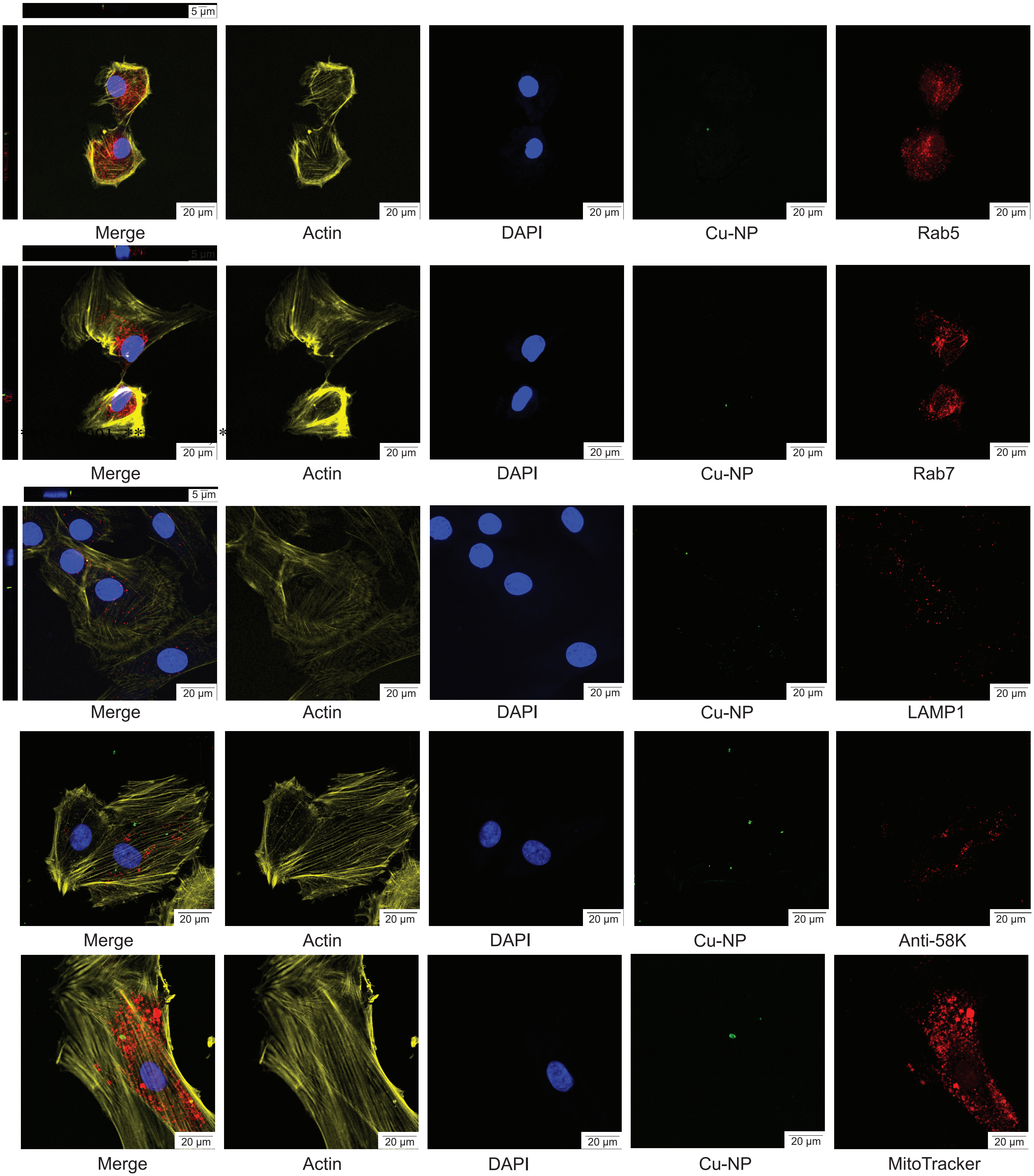

Figure S1: Confocal microscopy of *Ctr1*$^{-/-}$ H9c2 cells treated with Cu-NPs. Yellow: actin cytoskeleton; blue: nuclei; green: Cu-NPs; red: compartmnt specific stain (Rab 5: early endosomes; Rab 7: late endosomes; Lamp1: lysosomes; Anti-58K: Golgi body; MitoTracker: Mitochondria).

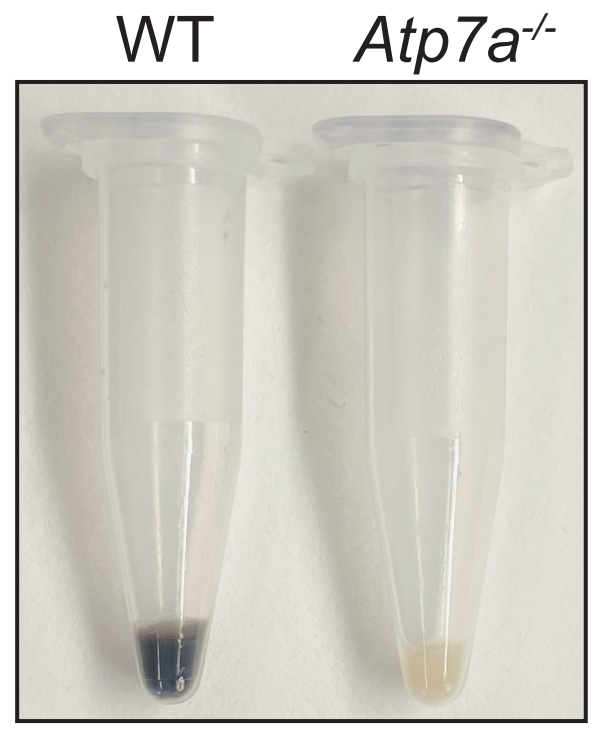

Figure S2: Wild-type (WT) and *Atp7a*$^{-/-}$ B16 cell pellets obtained after 48 hours of growth.

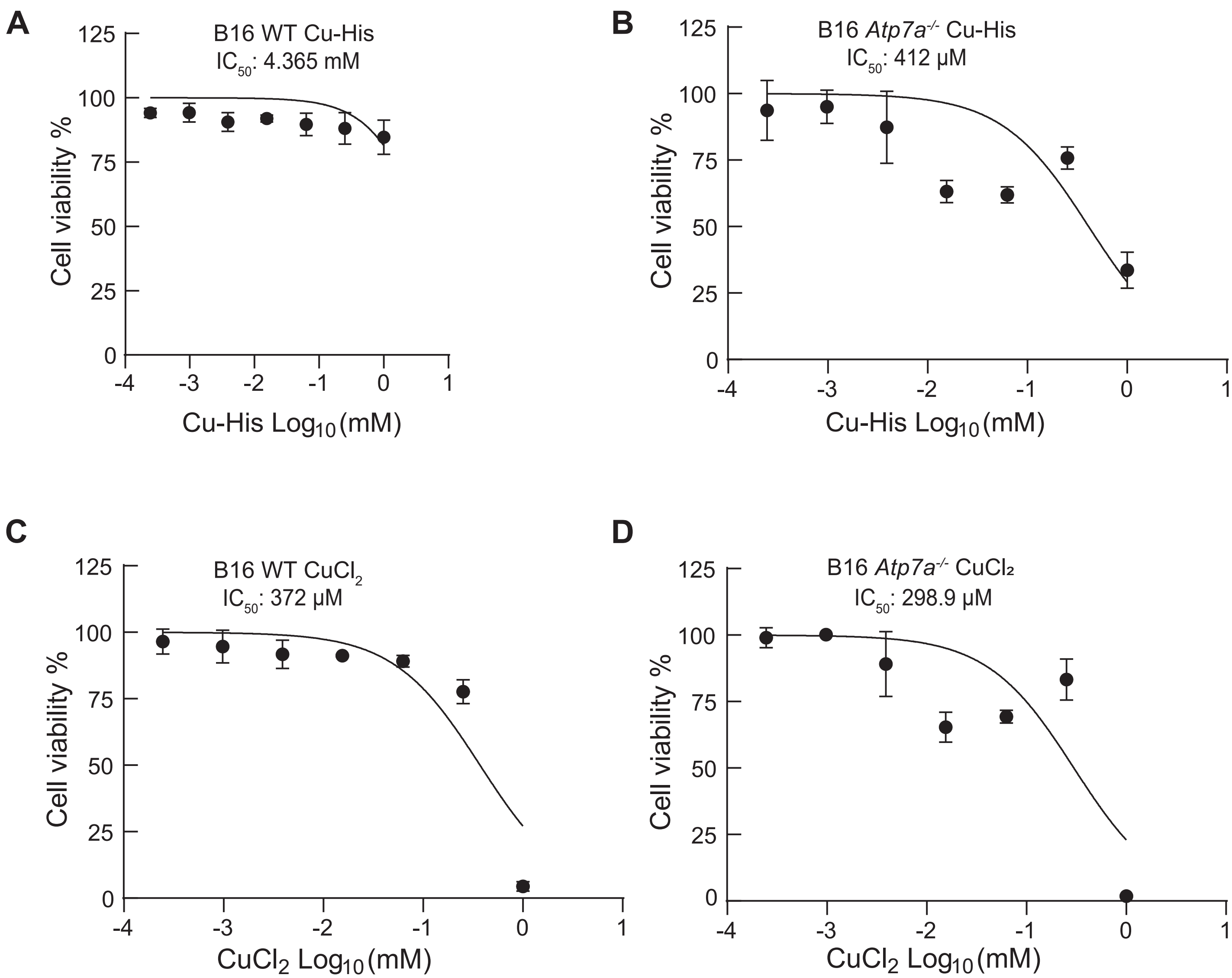

Figure S3: IC_{50} of Cu-Histidinate and $CuCl_2$ for WT (A, C) and *Atp7a*$^{-/-}$ (B, D) B16 cells. Cells were treated with increasing concentrations of each compound, and cell viability was measured using CellTitre-Glo after 48-hour treatment. Each data point represents three biological replicates and is expressed as mean ± S.D. (n = 3). For IC_{50} calculations, a log (inhibitor)vs. normalized response model was used.

Cu-NP Concentration (μg/ml)	Molarity (μM)
0.032	ND
0.16	2
0.8	5
2	13
4	34
10	54
20	164

Table S1: Concentrations of Cu-NPs (μM) measured using ICP-MS, n = 3.
